# Food choices and beliefs in adults with inflammatory bowel disease in Sweden

**DOI:** 10.1186/s12876-026-05300-8

**Published:** 2026-09-02

**Authors:** Sara Andersson, Katarina M Korwel, Klas Sjöberg, Katarina Pihl Lesnovska, Kevin Whelan, Charlotte RH Hedin, Alexandra Vulcan

**Affiliations:** 1 Medical Unit Clinical Nutrition, Theme Women’s Health and Allied Health Professionals, Karolinska University Hospital, Stockholm, Sweden; 2 Department of Medicine Solna, Karolinska Institutet, Stockholm, Sweden; 3 Department of Health, Medicine, and Caring Sciences, Linköping University, Linköping, Sweden; 4 Clinical Department of Gastroenterology and Hepatology, Region Östergötland, Linköping, Sweden; 5 Department of Clinical Sciences, Lund University, Malmö, Sweden; 6 Department of Gastroenterology, Skåne University Hospital, Malmö, Sweden; 7 Department of Nutritional Sciences, King’s College London, London, UK; 8 Centre for Digestive Health, Department of Gastroenterology, Dermatovenereology and Rheumatology, Karolinska University Hospital, Stockholm, Sweden

**Keywords:** Dietary beliefs, Food choices, IBD

## Abstract

**Objectives:**

Diet is a recognised risk factor for inflammatory bowel disease (IBD) and a management strategy. Concerns persist regarding the adverse effects of restrictive diets, including nutritional deficiencies and selective eating. The impact of IBD on patients’ beliefs and dietary choices remains poorly characterised, especially in different geographic contexts.

**Aim:**

To investigate dietary practices and beliefs in patients with IBD in Sweden, including variations by disease phenotype.

**Methods:**

A 42-item questionnaire on food choices and beliefs was distributed to all adult members of Sweden’s largest IBD-patient organization.

**Results:**

Of the 2,193 individuals invited, 738 (34%) responded, (Crohn’s disease (CD) 361 (49%); Ulcerative colitis (UC) 377 (51%)). IBD impacted thoughts about food in 71% (*n* = 521). A higher proportion of those with CD (254, 70%) than UC (231, 61%), reported changing their diet due to IBD (*p* = 0.009). A higher proportion of patients with CD (187, 52%), compared with UC (164, 43%), avoid certain foods in the belief that this can prevent flare-ups (*p* = 0.024). 42% (*n* = 311) of patients had not received dietary advice from health care providers, which was more common in UC (209, 55%) than CD (102, 28%), (*p* < 0.001).

**Conclusions:**

Beliefs about food and diet appear to influence dietary choices in IBD, many of whom report modifying their diet. This appears to be more pronounced among people with CD than UC, although the latter are less likely to receive dietary advice, highlighting potential disparities in access to dietary guidance.

**Supplementary Information:**

The online version contains supplementary material available at https://doi.org/10.1186/s12876-026-05300-8.

## Introduction

Inflammatory bowel disease (IBD) is a chronic, relapsing condition of the gastrointestinal tract comprising two major phenotypes: Crohn’s disease (CD) and ulcerative colitis (UC). IBD typically presents with symptoms such as abdominal pain, diarrhoea, fatigue, and weight loss [1] and can significantly impact quality of life (QoL) [2]. IBD has become a global disease, with a prevalence exceeding 0.3% in North America, Oceania, and many countries in Europe, while incidence continues to rise in newly industrialized nations [3]. In Sweden, the estimated prevalence of IBD is 0.65% [4] and data from a population-based birth cohort reported a cumulative incidence of 0.74% by early adulthood [5]. The aetiology of IBD is multifactorial, with diet hypothesized as one environmental risk factor [6]. Dietary choices are commonly used in the management of IBD, with patients frequently modifying their diets to control symptoms and prevent and treat disease flares, changes that are made either through personal experience or recommended by the clinical team [7]. Many individuals with IBD attribute a role for diet in the development or progression of their condition, influencing food choices and leading to strategies such as avoiding specific foods, following specialised diets, or adopting general healthy eating patterns [8, 9]. Restrictive dietary modifications may result in increasingly limited diets and, in some cases, contribute to selective eating behaviours, malnutrition and a negative impact on quality of life [10]. In a recent scoping review [11], 34 studies investigating this issue were identified, demonstrating that 56–91% of individuals with IBD modify their diet after diagnosis, either through some form of food avoidance (41–95%) or by attempting restrictive dietary behaviours. A few studies have also compared dietary behaviours between UC and CD [12–20].

These wide ranges observed across studies reflect wide variations in experience of individual patients and across different populations. Meanwhile, the factors associated with these dietary perceptions and behaviours have undergone limited examination due to small sample sizes and power to detect differences. Furthermore, most previous studies, including those in the scoping review, are also limited by being local to a specific unit rather than through nationwide sampling, and have restricted geographic coverage in different countries, including no previous data from Sweden. This underscores a clear knowledge gap that necessitates further research.

Altered dietary behaviours are not without consequences. Inadequate dietary intake may be associated with impaired food-related quality of life (FR-QoL). FR-QoL encompasses the psychosocial aspects of eating, including adequate nutrition, enjoyment of food, and social interactions around meals [21]. In individuals with IBD, impaired FR-QoL is common and has been associated with disease flares, increased frequency of flares, more severe symptoms, diminished IBD-specific QoL, and increased psychological distress, as well as lower intakes of some key nutritients [7, 22]. Despite the central role of diet in IBD management, dietary guidance remains inconsistent among health care providers [23], highlighting the need for further research into how dietary support can promote both clinical outcomes and FR-QoL.

The aim of this study was to examine dietary choices among Swedish patients with IBD and their perceptions of the impact of diet on disease and FR-QoL, and to compare these between CD and UC. Our study will help to identify specific needs that could inform future dietary support and clinical practice.

## Materials and methods

### Study population

The present study was an observational cross-sectional study conducted in adult individuals with IBD in Sweden between November to December 2024. All adult members (> 18 years) of Sweden’s largest nationwide patient association for individuals with IBD (Mag- och tarmförbundet) were invited to participate in the study, a total sampling frame of 2193 individuals. All individuals were eligible and we did not apply any exclusion criteria to encourage a representative sample. A 42-item questionnaire, hosted on Google Forms, was electronically distributed by Mag-tarmförbundet via email using its membership database. For participants who preferred not to complete the electronic questionnaire, a paper version was made available. Two reminder emails were sent, as this has been shown to increase response rates [24], which resulted in a twofold increase in the number of responses. Demographic data for the sampling frame were extracted after the study completion, at which time membership had increased marginally from 2193 to 2224.

### Development and design of the questionnaire

A study-specific questionnaire was developed to collect data from individuals with IBD on their dietary choices, reasons for these choices, and beliefs about their impact on disease outcomes and symptom management. The questionnaire was designed by a specialist gastroenterology dietitian and was informed by a comprehensive literature review to ensure relevance and completeness. The draft questionnaire was then refined after review by three expert dietitians. Prior to distribution, the questionnaire underwent informal assessment of readability and perceived relevance by two patients with IBD. The final questionnaire consisted of 42-items including a combination of multiple-choice questions, open-ended responses, and binary response options. Free-text responses were collected separately and were not included in the quantitative analysis presented in this study. A separate qualitative synthesis of these responses will be reported elsewhere. The food choice question (Q1) consisted of predefined response categories, including an “Other food choices” option that allowed participants to provide a free-text response, and participants were instructed to select the option that best described their predominant dietary pattern. The categories were not intended to capture all possible overlaps in dietary behaviours. For a summary of the questions analysed in this study, see Supplementary Table 1.

### Study variables

Demographic and disease-related data were collected through the questionnaire and included sex (male, female), year of birth, educational level (primary, secondary, post-secondary education), disease phenotype (CD, UC), disease location (small bowel, colon, rectum, don’t know) and disease duration (years). The second part of the questionnaire explored six main domains; dietary patterns and food choices, dietary modifications due to IBD, impact of IBD on food-related thoughts and social behaviour, beliefs about diet and disease, sources of dietary information and perceived role of diet compared with medication and use of supplements and alternative medicine.

### Statistical analysis

Descriptive analyses were used to present the overall results of the questionnaire for the total sample, including demographic and disease-related variables as well as responses to the food-related questions. These data were summarized using median (interquartile range, IQR) or n (%), depending upon data type.

Univariate analyses were conducted to examine differences between CD and UC. For categorical outcomes, Pearson’s chi-square test was used with results presented as n (%); for continuous variables, the Wilcoxon rank-sum test was used with values summarized as median (IQR). Multiple logistic regression analysis was used to examine associations between multiple independent variables (age, gender, diagnosis, disease duration, education) and a binary outcome. Model fit was assessed using the likelihood ratio chi-square test, and individual predictors were evaluated using Wald tests. Pseudo R² was reported as an indicator of explained variance. Odds ratios (OR) with 95% confidence intervals (CI) were calculated to estimate the strength and direction of associations.

Missing data were handled using available-case analysis (item-level exclusion), with participants excluded only from analyses for which relevant data were missing. The extent of missing data was minimal across questionnaire items, and any deviations in the number of observations are reported in the corresponding tables or text. Unless otherwise specified, analyses were based on complete responses from all 738 participants. Throughout, statistical significance was defined as *p* < 0.05. All statistical analyses were performed using STATA (version 19.5 SE-Standard edition; Statacorp LCC, College station, Tx, USA).

### Ethical considerations

The study was approved by the Swedish Ethical Review Authority (Dnr: 2024-04814-01). Participants were provided with written information about the study before participation. Participation was voluntary, confidential and anonymous. Informed consent was implied through voluntary completion of the survey. This study was performed in accordance with relevant guidelines and regulations, including the Declaration of Helsinki.

## Results

### Patient demographics

A total of 738 (34%) of the 2193 invited individuals completed the questionnaire. The patients included in the study were representative of the total eligible population regarding age, gender, and disease phenotype (Supplementary Table 2). Patient demographics are presented in Table 1. As indeterminate colitis only accounted for nine individuals, they were excluded from further analysis. There were no significant differences between patients with CD and UC in age, sex, or education. However, patients with CD had a longer disease duration than UC.

**Table 1:** Demographics and disease characteristics of the respondents (*N* = 738): comparison between CD and UC (Pearson’s Chi-squared test or Wilcoxson rank-sum (Mann-Whitney) test, p-value < 0.05)

Variable	Total (*N* = 738)	CD (*n* = 361)	UC (*n* = 377)	*p*-value ^a^
Age, years; median, (IQR)	61 (49–70)	61 (50–70)	60 (48–70)	0.215
Sex, female, n (%)	541 (73)	271 (75)	269 (71)	0.220
Self-reported disease location, n (%)				
Small intestine	250 (34)	243 (67)	7 (2)	<0.001
Colon	500 (68)	200 (57)	293 (78)	<0.001
Rectum	227 (31)	45 (12)	182 (48)	<0.001
Don’t know	13 (2)	5 (1)	8 (2)	0.579
Disease duration, years, median, (IQR)	23 (10–36)	27 (12–40)	20 (9–31)	**<0.001**
Unknown, n (%)	8 (1)	0	8 (2)	
Education, n (%)				0.532
Primary education	61 (8)	34 (9)	27 (7)	
Secondary education	263 (36)	128 (35)	135 (36)	
Post-secondary	414 (56)	199 (55)	215 (57)	

Values are presented as median (IQR) for continuous variables and n (%) for categorical

^a^ Pearson’s Chi-square test for sex, self-reported disease duration and education. Wilcoxson rank-sum (Mann-Whitney) test for age and disease duration

### Food choices, dietary patterns & supplementation

In total, a fifth stated that they followed a specific diet or eating pattern, with no significant difference between patients with CD and UC (Table 2). Significantly more patients with UC than CD stated they were omnivores. A low intake of vegetables, root vegetables, fruits, and berries was significantly more common in CD compared with UC. “Other food choices” was selected by a fifth of participants; these responses included free-text descriptions of more heterogeneous dietary patterns, which were not further categorised in the present quantitative analysis.

**Fig. 1:**
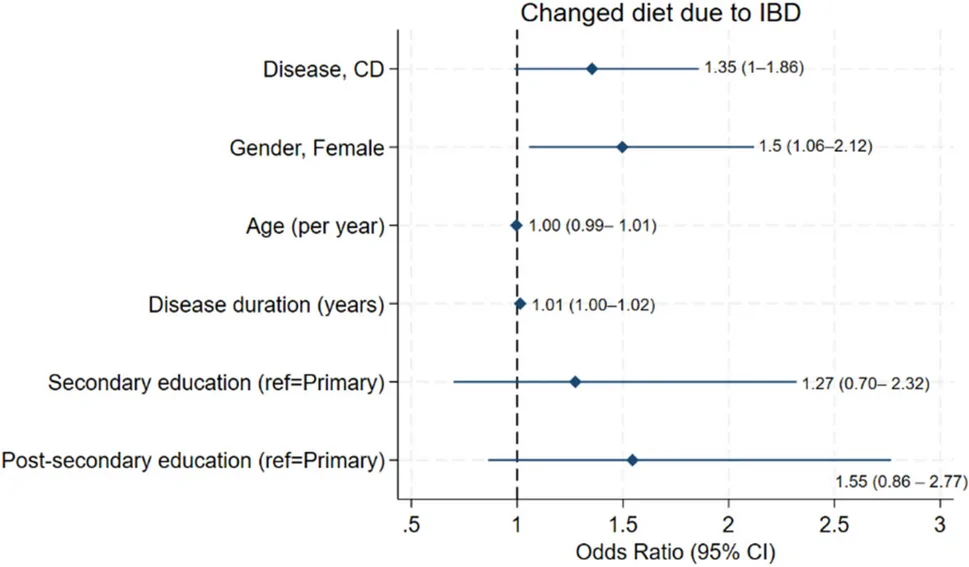
Forest plot of odds ratios (OR) and 95% confidence intervals for predictors of having changed diet due to IBD. Results are based on a multiple logistic regression model including disease type (CD vs. UC), sex (female vs. male), age (per year), disease duration (years), and education level. Education level was categorized as: 1 = primary education, 2 = secondary education, and 3 = post-secondary education

Two thirds reported changing their diet due to IBD which was significantly more common among patients with CD than with UC. In the multiple logistic regression analysis, females had significantly higher odds ratio for having changed their diet due to IBD compared with males. The overall model fit for this analysis was significant (*p* = 0.003). The full logistic regression model was statistically significant (LR χ² = 19.85) indicating that the predictors included in the model explain a statistically significant portion of the variance in dietary change behaviour among patients. The model achieved a Pseudo R² (McFadden) of 0.021 (Fig. 1, Supplementary Table 3).

**Fig. 2:**
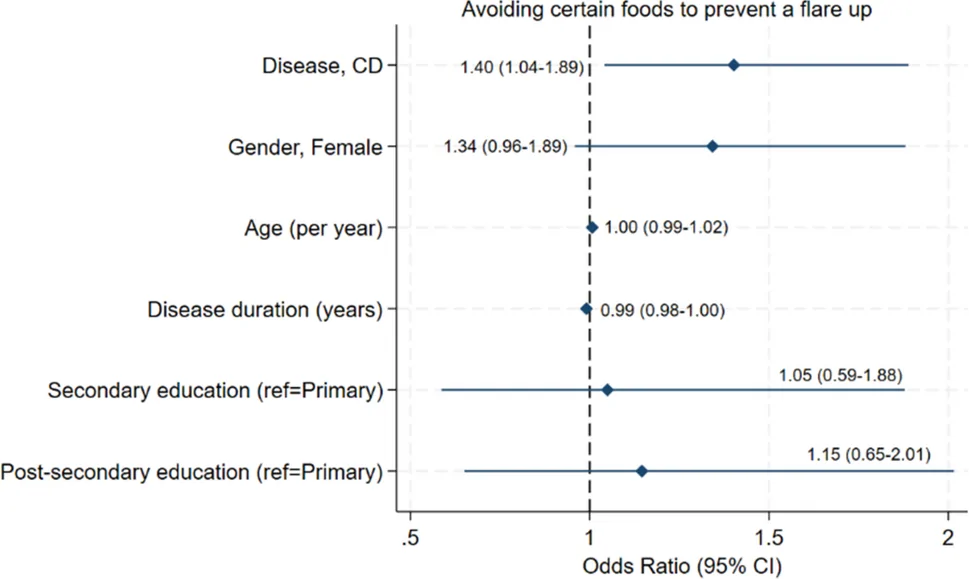
Forest plot of odds ratios (OR) and 95% confidence intervals for predictors of avoiding certain foods to prevent a flare up. Results are based on a multiple logistic regression model including disease type (CD vs. UC), sex (female vs. male), age (per year), disease duration (years), and education level. Education level was categorized as: 1 = primary education, 2 = secondary education, and 3 = post-secondary education

Half of respondents avoided certain foods to prevent a flare, a behavior more commonly reported among patients with Crohn’s disease than ulcerative colitis in univariate analyses. In the multiple logistic regression model adjusted for age, sex, disease duration, and education, the overall model did not reach statistical significance (LR χ² = 10.53, *p* = 0.104; McFadden’s Pseudo R² = 0.010), indicating limited explanatory power. Adjusted odds ratios are presented for completeness; however, given the lack of overall model significance, these estimates should be considered exploratory and interpreted with caution (Fig. 2, Supplementary Table 3). Additionally, a quarter of the patients avoided eating out or eating at others’ homes because of their condition, also more common in CD than in UC. A significantly greater proportion of patients with CD supplemented their diet with vitamin and/or mineral supplements compared with UC, while a significantly greater proportion of patients with UC took these supplements on their own initiative as compared with CD (Table 2).

**Table 2:** Food choices, dietary patterns and supplementations in IBD patients (*N* = 738): comparison between CD and UC (Pearson’s Chi-squared test, p-value < 0.05)

Variable Agreement with the statement, *n* (%)	Total (*N* = 738)	CD (*n* = 361)	UC (*n* = 377)	*p*-value ^a^
Option best describing their food choice				
Omnivore	495 (67)	221 (61)	274 (73)	**0.001**
Low intake of fruit, vegetables and berries	87 (12)	58 (16)	29 (8)	**< 0.001**
Excludes meat and poultry	11 (1)	4 (1)	7 (2)	0.400
Lakto-ovo-vegetarian	15 (2)	7 (2)	8 (2)	0.860
Other food choices	130 (18)	71 (20)	59 (16)	0.152
Follow a special diet	138 (19)	72 (20)	66 (18)	0.396
Changed the diet due to IBD	485 (66)	254 (70)	231 (61)	**0.009**
Avoid certain food to prevent flare up	352 (48)	187 (52)	164 (43)	**0.024**
Eat the same food as household members				0.089
Yes	355 (57)	167 (54)	188 (60)	
Partly/no	267 (43)	144 (46)	123 (40)	
Not applicable ^b^	116 (16)			
Avoid eating out or at others’ homes due to fear of symptoms	188 (25)	108 (30)	80 (21)	**0.007**
Takes vitamin- and/or mineral supplements				**< 0.001**
Yes, with prescription	236 (32)	164 (45)	72 (19)	
Yes, without prescription	251 (34)	102 (28)	149 (40)	
No	251 (34)	95 (26)	156 (41)	
Takes alternative medicine or supplements due to IBD	160 (22)	88 (24)	72 (19)	0.082

Values are presented as *n* (%)

^a^ Pearson’s Chi-square test

^b^ Statistical comparison did not include those individuals who indicated this was not applicable

### Beliefs and perceptions

**Fig. 3:**
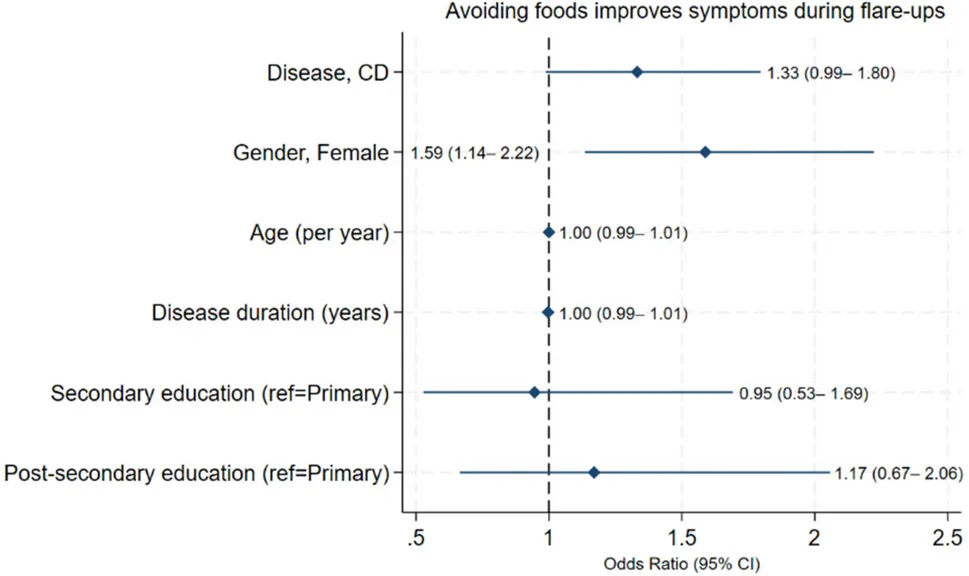
Forest plot of odds ratios (OR) and 95% confidence intervals for predictors of avoiding foods to improve symptoms during flare-ups. Results are based on a multiple logistic regression model including disease type (CD vs. UC), sex (female vs. male), age (per year), disease duration (years), and education level. Education level was categorized as: 1 = primary education, 2 = secondary education, and 3 = post-secondary education

Many patients indicated that IBD affect their thoughts about food, with no significant difference between UC and CD (Table 3). Significantly more patients with CD compared to UC believed that diet could cause a flare and that dietary changes could shorten it. Just over half believed that avoiding certain foods improved symptoms during flare-ups, with a significantly higher proportion in CD than in UC. In the multiple logistic regression model, female gender was significantly associated with higher odds of reporting symptom improvement during flares when avoiding certain foods (OR 1.59, 95% CI 1.14– 2.22). The overall model fit was significant (LR χ² = 13.94, *p* = 0.030), indicating that the included predictors contributed to explaining variation in the outcome (McFadden’s Pseudo R²=0.014) (Fig. 3, Supplementary Table 3). About half of the patients perceived diet as equally or more important than medication compared to those who considered diet less important or not important, and this perception did not differ significantly between CD and UC (Table 3).

### Dietary advice

**Fig. 4:**
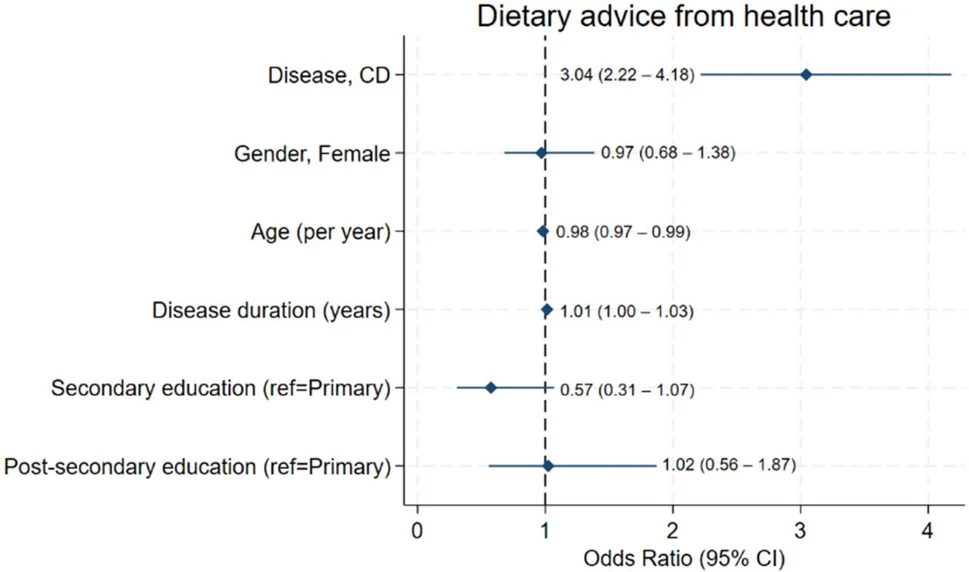
Forest plot of odds ratios (OR) and 95% confidence intervals for predictors of receiving dietary advice from health care. Results are based on a multiple logistic regression model including disease type (CD vs. UC), sex (female vs. male), age (per year), disease duration (years), and education level. Education level was categorized as: 1 = primary education, 2 = secondary education, and 3 = post-secondary education

In total, 460 (62%) had received dietary advice from various sources. Overall, 427 (58%) had received dietary advice from health care providers, and this was significantly more common among CD (259, 72%) than UC (168, 45%), (*p* < 0.001). In the multiple logistic regression model, CD was associated with threefold higher odds (OR = 3.04, 95% CI 2.22–4.18) of reporting dietary advice from health care providers, confirming the bivariate finding from the chi-square test. Age was negatively associated with the outcome, indicating that younger individuals were more likely to receive advice, whereas disease duration showed a positive association, with longer duration corresponding to a higher likelihood of receiving advice. The overall model fit was significant (LR χ² = 75.08, p = < 0.001) and explained a modest proportion of the variance (McFadden’s Pseudo R² = 0.076) (Fig. 4, Supplementary Table 3).

When patients were asked where they received information about which foods to avoid because of their IBD, 92% (682/738) responded to this question, while 8% (56/738) selected the response option ‘not applicable. Of the respondents, 75% (508/682) indicated that they rely on their own experience; with no significant difference between CD (266/682, 77%) and UC (242/682, 72%), (p = 0.086). 35% (240/682) reported receiving information from health care providers, which was significantly more common in CD (158/682, 46%) than in UC (82/682, 24%), (p < 0.001). Finally, 15% (109/682) stated that they receive information from social media or the internet; sources significantly more frequently reported by patients with UC (69/682, 20%) than CD (40/682, 12%), (p = 0.002).

**Table 3:** Beliefs and perceptions among IBD patients (*N* = 738): comparison between CD and UC (Pearson’s Chi-squared test, p-value < 0.05)

Variable Agreement with the statement, *n* (%)	Total (*N* = 738)	CD (*n* = 361)	UC (*n* = 377)	*p*-value ^a^
IBD affects their thoughts about food	521 (71)	266 (74)	255 (68)	0.060
Diet can cause a flare-up				**0.004**
Yes	183 (25)	104 (29)	79 (21)	
Maybe	309 (42)	156 (43)	153 (41)	
No	246 (33)	101 (28)	145 (38)	
Avoiding foods improves symptoms during flare-ups	394 (53)	207 (57)	187 (50)	**0.035**
Can shorten flares by changing diet ^b^		^b^		**0.042**
Yes	154 (21)	83 (23)	71 (19)	
Maybe	323 (44)	166 (46)	157 (42)	
No	260 (35)	111 (31)	149 (40)	
Diet is a cause of their IBD				0.199
Yes	60 (13)	24 (7)	36 (10)	
Maybe	252 (34)	132 (37)	120 (32)	
No	426 (58)	205 (57)	221 (59)	
Diet is ^b^		^b^		
Equally/more important than medication	367 (50)	185 (51)	182 (48)	0.398
Not/less important than medication	370 (50)	175 (49)	195 (52)	0.398

Values are presented as *n* (%)

^a^ Pearson’s Chi-square test

^b^ One missing response in the CD group (*n* = 360 for these items in CD subgroup analyses)

## Discussion

This is one of the largest studies assessing dietary choices and beliefs in IBD and, to our knowledge, the first quantitative questionnaire-based study in the Nordic region to compare differences between CD and UC within this geographical and cultural context. Both patients with CD and UC report restricting their food intake; for some outcomes, this was significantly more common among patients with CD, while for others no differences were observed. Living with IBD appears to influence patients’ perceptions of food, leading to social limitations and reduced FR-QoL. At the same time, there is a considerable lack of dietary guidance provided by health care professionals, particularly among patients with UC compared with CD, highlighting potential gaps in dietary counselling. Importantly, this study reflects self-reported perceptions and beliefs and does not allow conclusions about causal relationships between diet and disease activity.

Our study identified some novel findings: significantly more patients with CD reported avoiding foods to improve symptoms and believed that dietary changes could shorten flare duration, findings that, to our knowledge, have not previously been reported. These differences may partly be explained by variations in age and disease duration, as longer disease duration is often associated with more flareups and greater patient experience with dietary management. In addition, our multiple logistic regression analysis showed that women had higher odds of avoiding specific foods to improve symptoms, suggesting that sex-related behavioural or psychosocial factors may also influence dietary modification practices. This pattern is consistent with other research indicating that women are generally more likely to engage in symptom driven dietary adjustments when coping with gastrointestinal symptoms [25]. Furthermore, patients with CD were significantly more likely to use vitamin or mineral supplements, either self-initiated or prescribed. While a Polish study [14] reported overall supplement use of 45%, with higher prevalence among patients with CD (59%) compared to UC (35%, *p* < 0.05), our findings add nuance: patients with UC in our cohort were significantly more likely to take supplements on their own initiative. This may reflect differences in healthcare support or prescribing practices but may also be related to the generally lower risk of nutritional deficiencies in UC compared with CD, where small bowel involvement and malabsorption are more common. These observations highlight the need for tailored dietary counselling that addresses patient characteristics and ensures equitable access to evidence-based guidance.

In the present study, two-thirds reported having changed their diet due to IBD, a behaviour that was more common in CD than in UC. This contrasts with findings from a study conducted in India [16], where dietary changes were more frequent among patients with UC (88%) compared to CD (80%). Such discrepancies may reflect variations in study populations, cultural contexts, and methodological approaches. For example, that study [16] included younger respondents and shorter disease duration, both of which may be influential factors. Moreover, CD-related complications, which may also impact dietary beliefs and behaviours, often develop later in the disease course and may therefor further contribute to the differences observed.

In our study, a greater proportion of patients with CD compared to UC believed that diet could trigger disease flares and reported avoiding certain foods to prevent flare-ups. Similar patterns have been reported in a study in the United Kingdom (UK) [17]. Likewise, another study [14] reported that 91.7% of the patients with CD, compared with 67.6% of the patients with UC (*p* < 0.05), believed certain foods should be avoided to prevent disease recurrence. These results suggest a stronger perception among patients with CD regarding the perceived role of diet in symptom management and a greater tendency toward restrictive eating behaviours.

Several factors may explain why patients with CD express stronger beliefs about the dietary influence on disease activity and engage more frequently in food avoidance. CD is typically associated with a more variable and complicated disease course, including strictures, fistulas, and small bowel involvement, which may heighten patients’ awareness of the gastrointestinal consequences of specific foods. Moreover, because nutrient absorption occurs predominantly in the small intestine, the primary site of inflammation in many patients with CD, individuals with CD may be more inclined to attribute symptoms or flare-ups to dietary triggers. These clinical characteristics may therefore reinforce patients’ perception that diet is central to symptom management and contribute to more restrictive eating behaviours among patients with CD.

A substantial proportion of participants reported not receiving any dietary advice, a finding consistent with previous studies where between 25 and 70% of patients lacked such guidance [13–17]. Our results further show that fewer patients with UC received any dietary advice compared with those with CD, a finding that mirrors another study [13] in which 36% of patients with UC and 22% of patients with CD reported not receiving any dietary advice. This suggests potential disparities in care that warrant attention, particularly given that an equal proportion of individuals with CD and UC reported that IBD influences their thoughts about food. The absence of significant differences in this concern suggests that all patients, regardless of phenotype, require equitable access to evidence-based dietary guidance.

In the present study, many patients reported not receiving dietary advice from health care providers, a finding consistent with previous research. A study from the UK [17] observed that only 30% of patients obtained recommendations from non-medical sources such as the Internet, information leaflets, or other IBD patients. Qualitative data further illustrate this gap: semi-structure interviews [26] indicating that patients received dietary advice from their gastroenterologist at diagnosis, accompanied by statements such as ‘it doesn’t matter what you eat.’ Guidance from dietitians in the study varied substantially, ranging from general advice (‘eat everything in moderation’) to restrictive recommendations during flares (‘limit diet and increase energy intake’). Consequently, many patients expressed uncertainty about appropriate food choices during flares and the need for vitamins or mineral supplementation [26]. Another study from the UK [23] highlighted similar inconsistencies among health care professionals, noting that 61% recommended limiting specific foods to reduce the risk of a relapse, most commonly high-fibre items (19%), despite limited evidence supporting fibre restriction in remission. Current ESPEN guidelines [27] suggest that fibre may even confer protective effects against CD, although this is not yet firmly established. These findings highlight the need for standardized, evidence-based dietary guidance for all patients with IBD to reduce uncertainty and reliance on non-medical sources that may promote overly restrictive diets. Equally important, health care providers may benefit from training to deliver consistent, evidence-based recommendations, ensuring that patients receive accurate and practical advice.

In addition, the present study found that patients with UC more frequently reported relying on social media or internet sources for dietary information compared with patients with CD. This may be clinically relevant, as these sources vary considerably in quality and may expose patients to misinformation or non-evidence-based dietary advice. In the context of the observed disparities in access to dietary counselling between UC and CD patients, this finding may suggest that reduced exposure to structured professional guidance in UC could contribute to a greater reliance on informal information sources. Given that both UC and CD patients reported that IBD influences their thoughts about food to a similar extent, these results highlight the importance of ensuring equitable access to consistent, evidence-based dietary counselling across all IBD subtypes to reduce reliance on potentially unreliable online sources.

The influence of IBD on food-related quality of life (FR-QoL) was not directly measured using a validated instrument in this study; rather, it was inferred from questionnaire items related to dietary changes, food-related thoughts, and social eating behaviours. These findings therefore suggest a potential impact on aspects of FR-QoL, as many respondents reported altering their diet post-diagnosis and acknowledged that IBD affects their thoughts about food. Social impacts were also evident, with participants reporting avoidance of meals outside the home and not eating the same foods as other household members. These observations are consistent with previous research suggesting that IBD may be associated with impaired FR-QoL and social eating behaviours [8, 28]. One study also suggests that greater health engagement and food involvement may be associated with improved FR-QoL and fewer hospitalisations and relapse rates [28]. Future research should assess FR-QoL using validated instruments, such as the IBD-specific FR-QoL questionnaire [21], to better understand how dietary beliefs, behaviours, and restrictions influence food-related quality of life in people with IBD. Interventions that enhance health engagement and food involvement may therefore represent promising strategies to improve aspects of FR-QoL, highlighting an important area for future research and clinical practice.

### Strengths and limitations

This study has several notable strengths. First, it includes a large sample size of 738 respondents, providing a robust dataset for analysis and enabling meaningful subgroup comparisons between CD and UC. Its nationwide coverage enhances the generalizability of findings within Sweden. Importantly, this is the first study to explore dietary practices and beliefs among IBD patients in Sweden, addressing a regional knowledge gap. The patient-centred approach captures real-world perceptions and behaviours, which are clinically relevant for improving care.

Several limitations should be acknowledged. First, selection bias may have occurred, as the sampling frame consisted of all members of a patient organisation, potentially overrepresenting individuals who are more engaged or health conscious, as well as response bias whereby individuals with less typical experiences or perspectives may be more likely to choose to participate. The reliance on self-reported data introduces risks of recall bias and social desirability bias. Additionally, the questionnaire was not formally validated, which may affect reliability and should be considered when interpreting the findings. Some variability in how questions were understood or reported may have occurred, particularly for items relating to dietary beliefs and behaviours. Therefore, the reported estimates and subgroup comparisons should be interpreted with appropriate caution. In addition, the assessment of questionnaire readability and perceived relevance involved only two patients, which may have limited the breadth of patient input during questionnaire development. The response rate of 34% raises concerns about non-response bias, although demographic comparisons between respondents and non-respondents did not reveal significant differences.

Disease activity (remission versus active disease) was not assessed and therefore could not be accounted for in the analyses. This represents a potential source of residual confounding, as dietary behaviours are likely to differ between patients in remission and those with active disease, with previous research suggesting that patients with active disease may adopt more restrictive dietary patterns. [16] Consequently, this may have influenced the observed associations.

In addition, other clinical characteristics, including disease severity and Crohn’s disease behaviour (e.g., stricturing or penetrating phenotypes), were not available in the present dataset and could therefore not be included in the analyses. These factors may influence dietary practices, food avoidance behaviours, and beliefs regarding the role of diet in symptom management and disease activity and may partly explain the observed differences between patients with CD and UC. Consequently, the findings should be interpreted in the context of underlying clinical heterogeneity that was not captured in this study. Information on previous surgery was collected in the questionnaire but was beyond the scope of the present analyses and will be reported elsewhere. Given that previous surgery may influence dietary practices and food-related experiences, its potential impact on the observed differences between patient groups cannot be excluded.

Further, the consistently low McFadden pseudo-R² values across the regression models indicate limited explanatory power, suggesting that dietary beliefs and behaviours in IBD are likely shaped by a broader range of psychosocial, behavioural, and clinical factors not captured in the present analysis.

Further, the study sample consisted of 73% female participants, which may limit the generalisability of findings to male patients, particularly given the overrepresentation of female participants in the sample. This imbalance may also reduce the statistical power to detect sex-specific differences and should be considered when interpreting the observed associations between female sex and dietary modification behaviours.

The predefined response categories for food choice (Q1) may not have fully captured the complexity of participants’ dietary behaviours. In addition, free-text responses within the “Other” category were not included in the quantitative analysis, which may have led to some misclassification. Other limitations include the inability to follow up or clarify individual responses, the potential for duplicate participation, and the exclusion of members without email addresses.

## Conclusion

This survey suggests that living with IBD is associated with a strong focus on diet in symptom management and flare prevention. Participants with both CD and UC reported food-related concerns, dietary restrictions, and social eating limitations, with avoidance behaviours particularly common among those with CD. Patients with UC are less likely to receive dietary advice than those with CD, highlighting potential gaps in care and disparities in access to dietary counselling. These findings highlight the need to integrate structured, evidence-based nutritional counselling into routine IBD care for all patients. Tailored guidance may help reduce restrictive eating patterns, prevent nutritional deficiencies, and improve overall QoL.

Future research should measure FR-QoL specifically using a validated tool and should also evaluate the impact of structured dietary counselling on clinical outcomes and FR-QoL and prioritize high-quality intervention studies to establish evidence-based recommendations for different disease states, including flares, remission, strictures, and functional symptoms. Developing standardized guidelines grounded in robust clinical evidence will be essential to ensure safe, effective, and consistent dietary advice across all stages of IBD care.

## Supplementary Information

Below is the link to the electronic supplementary material.

Supplementary Material 1

## Data Availability

All data supporting the findings of this study are available within the paper and its Supplementary Information. The data underlying this article can be shared on reasonable request.

## Abbreviations

IBD: Inflammatory bowel disease
CD: Crohn’s disease
UC: Ulcerative colitis
QoL: Quality of life
FR-QoL: Food‐related quality of life
IQR: Interquartile range
OR: Odds ratios
CI: Confidence intervals
LR χ²: Likelihood-ratio chi-square test
UK: United Kingdom
ESPEN: European Society for Clinical Nutrition and Metabolism
