# Supplementary material for: Food choices and beliefs in adults with inflammatory bowel disease in Sweden
Source: BMC Gastroenterol. 2026 Sep 2;26:538. doi: 10.1186/s12876-026-05300-8 (PMC13540890; doi:10.1186/s12876-026-05300-8)
Supplement: Supplementary file 1 [file 12876_2026_5300_MOESM1_ESM.docx]

**Supplementary tables**

**Supplementary Table 1.** **Dietary Practices and Beliefs Questionnaire (Q1–Q15).** Summary of questions analysed in this study. These questions are a section of a larger questionnaire. The other sections will be analysed separately.

| **Q#** | **Question** | **Response options** |
| --- | --- | --- |
| 1 | Which option best describes your food choices? | ☐ Omnivore ☐ Lacto-ovo vegetarian ☐ Low intake of fruit, vegetables and berries ☐ Excludes meat and poultry ☐ Other food choices |
| 2 | Do you follow a specific diet or eating pattern? | ☐ Yes ☐ No |
| 3 | Do you take any form of vitamin and/or mineral supplement? | ☐ Yes, with prescription ☐ Yes, without prescription ☐ No |
| 4 | Do you take any alternative medicine or supplements because of your IBD? | ☐ Yes ☐ No |
| 5 | Have you changed your diet or food choices because of your IBD diagnosis? | ☐ Yes ☐ No |
| 6 | To reduce the risk of a flare-up, do you avoid certain types of foods that you would otherwise like to eat? | ☐ Yes ☐ No |
| 7 | Do you feel that avoiding certain foods improves your symptoms during a flare-up? | ☐ Yes ☐ No |
| 8 | Do you feel that changing your food choices during a flare-up can help shorten its duration? | ☐ Yes ☐ No |
| 9 | Do you feel that your IBD diagnosis affects your thoughts about food? | ☐ Yes ☐ No |
| 10 | Do you and the people you live with eat the same types of foods? | ☐ Yes ☐ Partly ☐ No ☐ Not applicable |
| 11 | Do you believe that your diet is one of the causes of your IBD? | ☐ Yes ☐ Maybe ☐ No |
| 12 | Do you think that diet can cause an IBD flare-up? | ☐ Yes ☐ Maybe ☐ No |
| 13 | Compared to your medication for IBD, how important do you think diet is for your well-being or improvement in your IBD? | ☐ Equally important ☐ More important ☐ Less important ☐ Not important |
| 14 | If you avoid any foods because of your IBD, where did you receive information about which foods to avoid? | ☐ Own experience ☐ Healthcare ☐ Social media ☐ Internet ☐ Relatives ☐ Patient association ☐ Alternative care ☐ Other option ☐ Not applicable |
| 15a | Have you ever received dietary advice because of your IBD? | ☐ Yes ☐ No |
| 15b | If yes, who or what has been your source of information? | ☐ Own experience  ☐ Healthcare ☐ Social media ☐ Internet ☐ Relatives  ☐ Patient association  ☐ Alternative care  ☐ Other option  ☐ Not applicable |

**Supplementary Table 2.** Characteristics of the eligible study population and of the respondents of the survey. Values are presented as median (IQR) for continuous variables and n (%) for categorical.

| Variable | Study pop. N= 2224 | Respondent N= 738 |
| --- | --- | --- |
| Age, median (IQR) | 59 (46-69) | 61 (49-70) |
| Gender |  |  |
| Women, n (%) | 1561 (70) | 541 (73) |
| Men n (%) | 663 (30) | 197 (27) |
| Missing n (%) | 0 (0) | 0 (0) |
| Phenotype |  |  |
| Crohn’s disease n (%) | 1104 (50) | 361 (49) |
| Ulcerative colitis n (%) | 1120 (50) | 377 (51) |
| Missing n (%) | 0 (0) | 0 (0) |

**Supplementary Table 3.** Multiple logistic regression analyses for food-related outcomes. Values are presented as odds ratios (OR) with corresponding 95% confidence intervals (95% CI) (Lower–Upper), p‑values, likelihood ratio chi-square (LR χ²), the probability of χ² (Prob > χ²), and McFadden’s Pseudo R².

| Outcome (dependent variable), n=730* | Predictor | OR | 95% CI (Lower–Upper) | p-value | LR χ² | Prob > χ² | Pseudo R² |
| --- | --- | --- | --- | --- | --- | --- | --- |
| Changed diet due to IBD |  |  |  |  | 19.85 | 0.003 | 0.021 |
|  | Disease, CD | 1.35 | 1.00–1.86 | 0.060 |  |  |  |
|  | Gender, female | 1.50 | 1.06–2.12 | 0.023 |  |  |  |
|  | Age, per year | 1.00 | 0.99– 1.01 | 0.599 |  |  |  |
|  | Disease duration, years | 1.01 | 1.00–1.02 | 0.015 |  |  |  |
|  | Education, ref. primary education |  |  |  |  |  |  |
|  | - Secondary | 1.27 | 0.70– 2.32 | 0.427 |  |  |  |
|  | - Post-secondary | 1.55 | 0.86 – 2.77 | 0.143 |  |  |  |
| Avoiding certain foods to prevent flares |  |  |  |  | 10.53 | 0.104 | 0.010 |
|  | Disease, CD | 1.40 | 1.04– 1.89 | 0.026 |  |  |  |
|  | Gender, female | 1.34 | 0.96– 1.89 | 0.087 |  |  |  |
|  | Age, per year | 1.00 | 0.99– 1.02 | 0.230 |  |  |  |
|  | Disease duration, years | 0.99 | 0.98– 1.00 | 0.106 |  |  |  |
|  | Education, ref. primary education |  |  |  |  |  |  |
|  | - Secondary | 1.05 | 0.59– 1.88 | 0.870 |  |  |  |
|  | - Post-secondary | 1.15 | 0.65– 2.01 | 0.637 |  |  |  |
| Believe avoiding foods improves symptoms during flare-ups |  |  |  |  | 13.94 | 0.030 | 0.014 |
|  | Disease, CD | 1.33 | 0.99– 1.80 | 0.060 |  |  |  |
|  | Gender, female | 1.59 | 1.14– 2.22 | 0.007 |  |  |  |
|  | Age, per year | 1.00 | 0.99– 1.01 | 0.991 |  |  |  |
|  | Disease duration, years | 1.00 | 0.99– 1.01 | 0.585 |  |  |  |
|  | Education, ref. primary education |  |  |  |  |  |  |
|  | - Secondary | 0.95 | 0.53– 1.69 | 0.851 |  |  |  |
|  | - Post-secondary | 1.17 | 0.67– 2.06 | 0.586 |  |  |  |
| Dietary advice from health-care professionals |  |  |  |  | 75.08 | <0.001 | 0.076 |
|  | Disease, CD | 3.04 | 2.22 – 4.18 | <0.001 |  |  |  |
|  | Gender, female | 0.97 | 0.68 – 1.38 | 0.870 |  |  |  |
|  | Age, per year | 0.98 | 0.97 – 0.99 | 0.006 |  |  |  |
|  | Disease duration, years | 1.01 | 1.00 – 1.03 | 0.016 |  |  |  |
|  | Education, ref. primary education |  |  |  |  |  |  |
|  | - Secondary | 0.57 | 0.31 – 1.07 | 0.080 |  |  |  |
|  | - Post-secondary | 1.02 | 0.56 – 1.87 | 0.942 |  |  |  |

Notes: Coding; Diagnosis: 1 = Crohn's disease (CD), 0 = ulcerative colitis (UC). Sex: 1 = female, 0 = male. Education level: Primary education is the reference.

* Eight participants were excluded from the regression analyses due to missing data in one or more variables included in the models.
